# Prevalence and imaging features of nephrogenic systemic fibrosis at two large medical centers

**DOI:** 10.1186/1532-429X-11-S1-O57

**Published:** 2009-01-28

**Authors:** Martin R Prince, Honglei Zhang, Michael F Morris, Jennifer L MacGregor, Yang Zhang, Marc E Grossman, Jeffrey Silberzweig, Robert L DeLapaz, Henry J Lee, Cynthia M Magro, Anthony M Valeri, David N Silvers, Joan C Prowda

**Affiliations:** 1 Weill Medical College of Cornell University, New York, NY USA; 2 Columbia University, New York, NY USA

**Keywords:** Acute Renal Failure, Calcinosis, Nephrogenic Systemic Fibrosis, Hyperphosphatemia, Gadolinium Base Contrast Agent

## Introduction

Nephrogenic Systemic Fibrosis (NSF) is a rare disease, seen in patients with severe renal impairment, that has garnered increased interest among radiologists due to reports of its association with gadolinium based contrast agents (GBCA).

## Purpose

To determine the prevalence and illustrate the spectrum of imaging findings with photographic and histopathologic correlation of nephrogenic systemic fibrosis (NSF) in patients undergoing gadolinium based contrast agent (GBCA) enhanced MRI and associated risk factors.

## Methods

With Institutional Review Board approval (informed consent not required) medical records from two hospitals were retrospectively reviewed to identify all cases of biopsy-confirmed NSF and all patients receiving GBCA administration from 1/1/1997 to 6/30/2007. Prevalence of NSF was calculated for patients receiving standard dose GBCA, high dose GBCA as well as in subgroups of patients with renal impairment. Imaging studies of these patients were reviewed to correlate with photographic and histopathologic findings.

## Results

Fifteen patients had NSF following GBCA enhanced MR. All had an estimated glomerular filtration rate (eGFR) < 30 m L/min and 11 had acute renal failure or acute deterioration of chronic renal failure. NSF prevalence following GBCA without screening for renal function was 0/74124 for standard dose and 15/8997 (0.17%) for high dose (p < 0.001). The high dose prevalence increased to 0.4% for chronic hemodialysis patients and 8.8% for patients with eGFR < 15 mL/min but not on dialysis (p < 0.001). NSF prevalence in patients with acute renal failure given high dose GBCA during rising serum creatinine was 19% (11/58) when dialysis was delayed by > 2 days. More NSF patients had pro-inflammatory events, lower pH, younger age, lower eGFR, elevated serum phosphate and increased delay between GBCA injection and dialysis compared to patients without NSF.

NSF has a variable appearance on routine imaging studies. Plain radiographs can demonstrate joint contractures, skin thickening and possibly cutaneous calcinosis; ultrasound may show thickening and edema of the cutis, particularly in the breast; CT may show skin thickening and infiltration of subcutaneous tissues; MRI may show increased signal on fluid sensitive sequences in the skin, subcutaneous tissues, and extremity musculature; bone scintigraphy may show diffuse soft tissue uptake in the extremities.

## Conclusion

For patients with eGFR < 15 mL/min, hemodialysis is protective. For patients with eGFR < 30 mL/min receiving high dose GBCA, acute renal failure, delay in dialysis after GBCA injection, pro-inflammatory events and hyperphosphatemia were associated with increased NSF risk. Features of NSF may be evident on the patient's skin as well as on routine imaging studies although these imaging findings are nonspecific and more likely to occur with other diseases.

